# Bench to Bedside: Stability Studies of GMP Produced Trastuzumab-TCMC in Support of a Clinical Trial

**DOI:** 10.3390/ph8030435

**Published:** 2015-07-29

**Authors:** Diane E. Milenic, Kwamena E. Baidoo, Martin W. Brechbiel

**Affiliations:** Radioimmune & Inorganic Chemistry Section, Radiation Oncology Branch, Center for Cancer Research, National Cancer Institute, National Institutes of Health, Bethesda, MD 20892, USA; E-Mails: baidook@mail.nih.gov (K.E.B.); martinwb@mail.nih.gov (M.W.B.)

**Keywords:** monoclonal antibody, Pb-212, HER2, trastuzumab, radioimmunotherapy, FDA, stability

## Abstract

The first-in-human phase 1 clinical radioimmunotherapy (RIT) trial with ^212^Pb-1,4,7,10-tetraaza-1,4,7,10-tetra-(2-carbamoylmethyl)-cyclododecane-trastuzumab (^212^Pb-TCMC-trastuzumab) was completed in October 2014 as a joint effort at the University of Alabama (UAB) and the University of California San Diego Moores Cancer Center. The preliminary reports indicate that after five dose-levels of intraperitoneally administered ^212^Pb-TCMC-trastuzumab, patients with carcinomatosis experienced minimal agent-related toxicity. This report presents the data accumulated to date on the stability of the clinical grade, produced according to current good manufacturing practices (cGMP), TCMC-trastuzumab conducted in support of that clinical trial. Of the eleven tests performed with the cGMP TCMC-trastuzumab all but one remained within specifications throughout the 5 year testing period. The protein concentration varied by 0.01 mg/mL at 48 months. Two other assays, ion-exchange high performance liquid chromatography (IEX-HPLC) and a competitive radioimmunoassay (RIA) indicated that the cGMP TCMC-trastuzumab integrity may be changing, although the change thus far is within specifications. Subsequent stability testing will confirm if a trend has truly developed. The cGMP TCMC-trastuzumab was also evaluated for tolerance to higher temperatures and the potential of storage at −80 °C. The immunoconjugate proved stable when subjected to the lower temperatures and to multiple freeze-thaw cycles. The size exclusion (SE) HPLC analysis of the ^203^Pb-TCMC-trastuzumab was the only indicator that cGMP TCMC-trastuzumab may be sensitive to storage at 37 °C for 3 months.

## 1. Introduction

Pharmaceutical companies have taken a reinvigorated notice of therapeutic monoclonal antibodies (mAb) in the past decade and this continues to be a burgeoning field of product investigation and development. A casual perusal of magazines or viewing of the evening news one may see an advertisement for a mAb-based drug treating diseases such as psoriasis, Crohn’s disease or rheumatoid arthritis. By November 2014, 47 approved mAb products were on the market in the United States or Europe [1,2]. In 2014, of 41 new molecular entities and therapeutic biological products approved, 6 were mAb [1,2]. In parallel, recent approval by the Food and Drug Administration (FDA) of the first α-radiation drug, Xofigo (Bayer, NJ, USA), has been granted. This singular approval has prompted significant interest in targeted α-radiation therapies.

The road from bench to bedside with a therapeutic monoclonal antibody (mAb) is not for the faint of heart. From the start of Phase 2 trials, it is estimated to take ~seven years for a mAb to gain FDA approval and enter the market [1,2,3,4]. As a source for α-particle radiation, ^212^Pb can be exploited as an *in vivo* generator for ^212^Bi. The strategy traverses the logistical difficulties and time constraints of working directly with the shorter half-life (60.6 min) daughter radionuclide, ^212^Bi. This strategy as well as the numerous studies that this laboratory had published on the efficacy of ^212^Pb-TCMC-trastuzumab RIT attracted the attention of the French company, Areva Med LLC (Bethesda, MD, USA), which made first contact in 2007 [5,6,7,8,9]. Under the auspices of a Collaborative Research and Development Agreement (CRADA) approved in 2008, the translation of the *in vitro* and *in vivo* studies conducted by the Radioimmune & Inorganic Chemistry Section (RICS) to a clinical setting was initiated.

In 2009, following the rules of Good Manufacturing Practice, bulk clinical grade TCMC-trastuzumab (cGMP TCMC-trastuzumab) was produced under contract, tested and released for vialing; the validation assays were performed and thereafter stability testing was initiated as per FDA requirements. In January of 2011, Areva Med LLC acquired FDA-approval for the Investigational New Drug (IND) application and in July accrual began for the phase 1 human clinical study at UAB. The trial (NCT01384253) was designed to determine the potential toxicity of ^212^Pb-TCMC-trastuzumab, define its dose-limiting toxicities and to evaluate any anti-oncogenic effects in patients with intraperitoneal carcinomatosis. Patients accrued for the study were those with HER-2 positive tumors (e.g., ovarian, pancreatic, colon, gastric, endometrial, or breast) that scored either positive by immune-histo-chemistry (IHC ≥ 1+), demonstrated HER-2 amplification by *in situ* hybridization in more than 10% of the cells, or exhibited elevated serum HER-2 levels (≥15 ng/mL). The IHC scoring of 1+ is the equivalent of 92,400 ± 12,000 HER2 receptors on a tumor cell surface [10]. The last treatment cohort was completed in December, 2014. Minimal toxicity was reported for the first five cohorts (7.4 to 21.1 MBq/m^2^) with a follow-up of 4 months to 1 year [3,4]. The cGMP ^212^Pb-TCMC-trastuzumab was administered via an intraperitoneal catheter; the limited amount of radioactivity that redistributed into the circulating blood was eliminated via renal excretion. Imaging via whole body γ-scintigraphy indicated no specific uptake in the major organs.

The purpose of this report is to present the data accumulated to date on the stability of the cGMP TCMC-trastuzumab conducted in support of the clinical trial. The *in vitro* analysis of the cGMP TCMC-trastuzumab reported herein includes (1) visual inspection of the vialed product, (2) assessment of the integrity/purity of the immunoconjugate (IC) before and after radiolabeling with ^212^Pb, (3) determination of the product concentration variance versus the proportion of the conjugated TCMC chelate, and (4) evaluation of immunoreactivity before and after radiolabeling with ^212^Pb. Additionally, the stability of cGMP under stressed storage conditions at 25 °C and 37 °C was assessed. Finally, the tolerance of the cGMP TCMC-trastuzumab to storage at −80°C and subsequent freeze-thaw cycles was investigated.

## 2. Results

### 2.1. Visual Inspection

A mAb such as trastuzumab (Herceptin; Genentech Inc., South San Fransico, CA, USA) may be well characterized; however, conjugation of a mAb with a chelate creates a new molecule which then requires rigorous characterization. The final formulation of the cGMP TCMC-trastuzumab was in 0.15 M ammonium acetate (pH 7.0) at 5.25 mg/mL and stored at 2–8 °C. For testing purposes, 1–2 vials were used at each time point. Each vial was first visually inspected as it was removed from storage. No turbidity or particulates were noted in any of the vials removed for testing.

**Figure 1:**
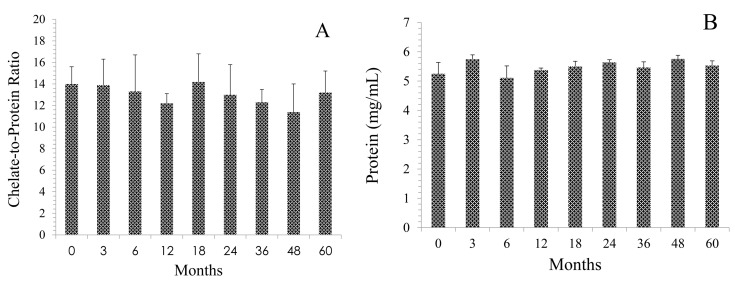
Effect of storage at 2–8 °C on the protein and chelate content of the current good manufacturing practices (cGMP) TCMC-Trastuzumab over a 60 month period. Panel **A**: Protein concentration was determined by the Lowry method using a BSA standard. Values are the average of three determinations and the error bars represent the standard deviation. Panel **B**: The molar ratio of chelate to protein was determined using the spectrophotometric assay based on the transchelation between Pb(II) and arsenazo (III). Values are the average of two experiments and the error bars represent the standard deviation.

The protein concentration was determined by the Lowry method using a bovine serum albumin (BSA) standard [11]. At the time of vialing (T0) the concentration of the cGMP TCMC-trastuzumab was 5.25 mg/mL. As shown in Figure 1A, over the five year study period, the protein concentration ranged from 5.1 to 5.76 mg/mL which averages to 5.46 ± 0.23 mg/mL. With the exception of the 48 month time point (5.76 mg/mL), the cGMP TCMC-trastuzumab concentration remained within specifications (4.75–5.75 mg/mL). The chelate-to-protein (C/P) ratio of the cGMP TCMC-trastuzumab also remained in compliance (14 ± 3). The C/P ratio ranged from 11.4 to 16.9 (13.1 ± 0.9) with no evidence of a trend over 5 years of testing (Figure 1B).

### 2.2. Integrity of cGMP TCMC-Trastuzumab

#### 2.2.1. Sodium Dodecyl Sulfate-Polyacrylamide Gel Electrophoresis (SDS-PAGE)

**Figure 2:**
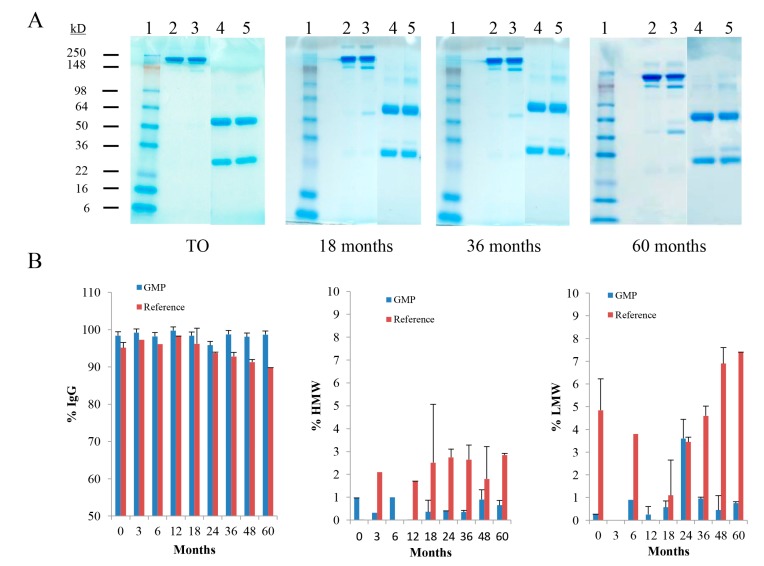
Analysis of cGMP TCMC-trastuzumab integrity and purity. Over a 60 month period the cGMP TCMC-trastuzumab was analyzed at T0, 3, 6, 12, 18, 24, 36, 48 and 60 months using SDS-PAGE and by SE-HPLC. Panel **A**: Representative images spanning the study period are shown with cGMP in Lanes 2 (non-reduced) and Lane 4 (reduced); the Reference TCMC-trastuzumab in Lanes 3 (non-reduced) and Lane 5 (reduced). Lane 1 contains the molecular weight standard. Panel **B**: The individual graphs from the SE-HPLC data depict the percentage of the IgG at ~15 min, the percentage of HMW species at ~12.6 min for monitoring of aggregate formation and the percentage of LMW species at ~21.4 min as an indicator of degradation. Values are the average of two experiments and the error bars represent the standard deviation.

The integrity and purity of the cGMP TCMC-trastuzumab was evaluated by SDS-PAGE, SE-HPLC (2.2.2.) and IEX-HPLC (2.2.3.). Representative gels spanning 5 years of testing the cGMP TCMC-trastuzumab are shown in Figure 2A. The major band of intact cGMP TCMC-trastuzumab (Lane 2) is evident at the expected Mr of ~148 kD with minor bands above and below it, a typical pattern observed with the majority of antibodies. Under reducing conditions using β-mercaptoethanol, the heavy and light chains are visualized with the coomassie stain (Lane 4). The appearance and patterns of the bands do not show any differences between the initial study (T0) and the 60 month evaluation. In contrast, some degradation is apparent over time in the Reference TCMC-trastuzumab which was generated in the RICS laboratory in parallel to the contract cGMP material (Lanes 3 and 5). At 18 months, there is the appearance of a band at ~40 kD that increases with intensity in the subsequent gels.

#### 2.2.2. Size-Exclusion High Performance Liquid Chromatography (SE-HPLC)

The SE-HPLC analysis corroborates the data of the SDS-PAGE. In Figure 2B, there is minimal variance in the % species that elutes at ~15 min, consistent with the elution time of an antibody under the conditions that the samples are applied and eluted. Meanwhile, there is a time-dependent decrease in the percentage of the Reference that is associated with immunoglobulin (IgG). Concomitantly, there is an increase in both the percentage of low molecular weight (LMW) and high molecular weight (HMW) species in the reference. The increase in the HMW species would be indicative of the formation of aggregates while the LMW species suggests that some degradation of the Reference is occurring. The presence of the HMW species in the Reference ranges from 1.7% to 2.9% with no time-dependent trend evident. The LMW entity ranges comprises 1.1% to 7.4% of the product. More importantly, the percentage of the LMW species shows an obvious trend upwards starting at the 24 month evaluation. Some HMW and LMW species are evident in the cGMP TCMC-trastuzumab preparation; however, they are low, with the exception of the 24 month analysis, and are not increasing with time. No LMW entity was in either the cGMP TCMC-trastuzumab or Reference at the 3 month evaluation or at 12 months in the Reference. Likewise, a HMW species was not present in the Reference material at the 0 and 6 month tests nor was any noted at 12 months in the cGMP TCMC-trastuzumab.

#### 2.2.3. Ion-Exchange High Performance Liquid Chromatography (IEX-HPLC)

**Figure 3:**
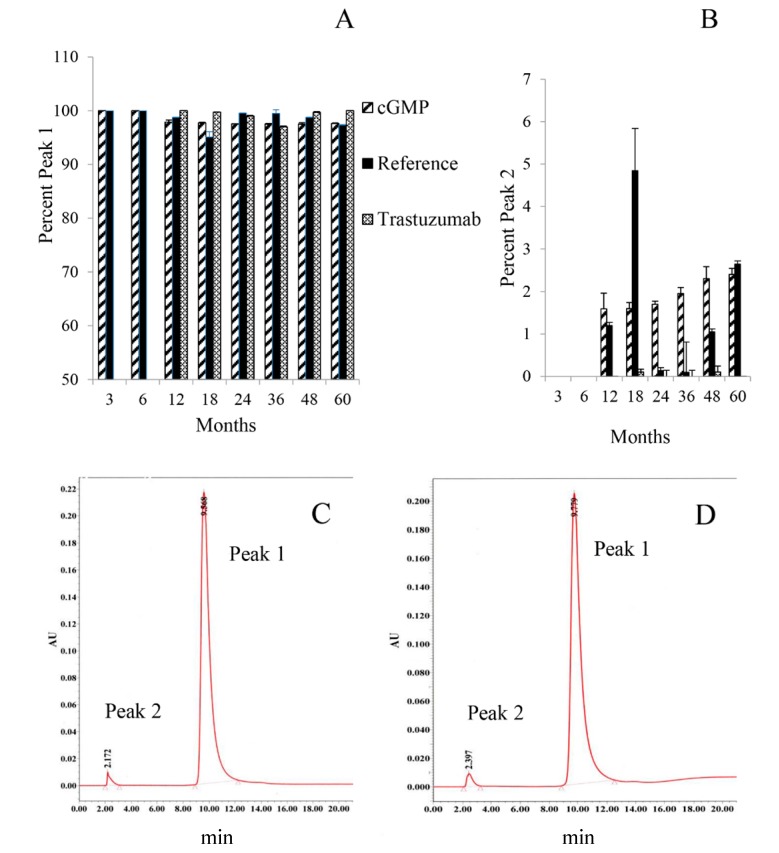
Ion-exchange (IEX) HPLC analysis of cGMP TCMC-trastuzumab. The IEX-HPLC a charge-based assay, serves as an additional test to assess the integrity of the cGMP TCMC-trastuzumab. After loading the sample on the column in 10 mM sodium phosphate (pH 7.2), a gradient of 0–100% 1 M NaCl gradient was begun from 5–20 min. Panel **A**: The cGMP TCMC-trastuzumab eluted at ~9.8 min (Peak 1). Panel **B**: A second peak (Peak 2) was observed at ~2.5 min representing material that was not retained on the column. Values are the average of two experiments and the error bars represent the standard deviation. Panel **C** and **D**: The IEX-HPLC chromatograms of the 24 month and 60 month evaluation, respectively, of cGMP TCMC-trastuzumab.

The IEX-HPLC served as a charged-based assay requested by the FDA. Incorporation of this assay into the stability tests did not occur until the 12 month time point. The cGMP TCMC-trastuzumab (150 µg) was applied to a column equilibrated in 10 mM sodium phosphate (pH 7.2), a gradient of 0–100% 1 M NaCl gradient was begun from 5–20 min. The cGMP TCMC-trastuzumab was eluted from the column at ~9.8 min. The results (Figure 3A) indicate that the behavior of the cGMP TCMC-trastuzumab remains virtually unchanged throughout the period studied with 97.3%–100% of the cGMP TCMC-trastuzumab eluting consistently from the column. With that stated, at 12 months, an additional peak (Peak 2) was detected at 2.5 min implicating that some material was not being retained by the column during the first 5 min of loading the sample (Figure 3B). In addition, Peak 2 did increase with time from 1.6% to 2.4% over 12 to 60 months, respectively. Peak 2 was also present in the Reference TCMC-trastuzumab, ranging from 0.1% to 4.9% with no trend evident over time. The peak was not present in the unmodified trastuzumab that was used as an additional reference in these studies.

### 2.3. cGMP TCMC Radiolabeling and Analysis of Immunoreactivity Before and After Radiolabeling

#### 2.3.1. *In Vitro* Analysis of cGMP ^212^Pb-TCMC-Trastuzumab

The data outlined in Table 1 presents the analysis of the cGMP ^212^Pb-TCMC-trastuzumab product. Radiolabelling efficiency of the cGMP TCMC-trastuzumab with ^212^Pb ranged from 92.2% to 99.6% using the instant thin layer chromatography (ITLC) assay [12] to determine the amount of free chelate in the final product. Until the 60 months, all time points were found to have 0.6% or less free chelate. At the 60 month stability testing, the amount of free chelated had increased to 1.6%. The purity of the ^212^Pb radiolabeled product, as determined by SE-HPLC, was 100% at all time points tested with the exception of the 24 and 60 month radiolabellings. At these two time points, the purity of the cGMP ^212^Pb-TCMC-trastuzumab was 91.5% and 90.2%, respectively. The specific activity of the ^212^Pb-TCMC-trastuzumab ranged from 2.8 to 6.0 mCi/mg.

**Table 1:** Analysis of cGMP TCMC-trastuzumab following labeling with ^212^Pb.

Months	% Efficiency	% Free Chelate	Specific Activity (mCi/mg)	% Purity (SE-HPLC)
0	92.2	0.6	3.2	100.0
3	92.2	0.2	3.3	ND
6	94.8	0.1	2.8	100.0
12	99.6	0.4	3.2	100.0
18	99.6	0.4	4.6	100.0
24	98.0	0.4	4.0	91.5
36	98.8	0.4	5.1	100.0
48	93.5	0.6	3.1	100.0
60	98.2	1.6	6.0	90.2

#### 2.3.2. Immunoreactivity of cGMP TCMC-Trastuzumab

Finally, the immunoreactivity of the cGMP ^212^Pb-TCMC-trastuzumab was evaluated using two RIA formats (Table 2). The first, a competition RIA, was performed with the cGMP TCMC-trastuzumab to ensure that the modification of trastuzumab with the chelate and subsequent storage at 2–8 °C did not alter its ability to bind to HER2. All assays included the Reference TCMC-trastuzumab and an anti-CD33 mAb, HuM195, as a negative control. Unmodified trastuzumab was not included in the competition RIAs performed at T0, 3 and 6 months. The cGMP TCMC-trastuzumab exhibited greater inhibition of the ^125^I-trastuzumab binding with the HER2 antigen (chimeric ERB2/Fc) for the first 4 years of testing (Table 2). In fact, less of the cGMP TCMC-trastuzumab was required to attain 50% inhibition than either the reference TCMC-trastuzumab or the unmodified trastuzumab. At the 60 month time point, however, the pattern was reversed in that more of the cGMP TCMC-trastuzumab was needed to obtain the 50% inhibition (50 ng/50 µL) versus 35 and 15 ng/50 µL for the TCMC-reference and the unmodified trastuzumab, respectively (Figure S1). Due to this result, the competition RIA was repeated using a new vial and fresh reagents with similar results being obtained (Figure S2).

**Table 2:** Immunoreactivity of the cGMP TCMC-trastuzumab immunoconjugate.

Months	Competition			Immunoreactivity (% Bound)					
				Specific	Non-Specific	Specific	Non-Specific	Adjusted % Bound	
	GMP	Ref	ROB	GMP	GMP	Ref	Ref	GMP	Ref
0	18.0 ^a^	20	ND ^b^	73.1	4.8	72.8	3.0	68.3 ^c^	69.8
3	3.0	3.2	ND	76.2	10.8	75.2	14.7	65.4	60.5
6	2.6	3.1	ND	57.8	12.6	67.5	11.4	45.2	56.1
12	1.2	2.9	2.9	62.2	6.4	59.9	3.2	55.8	56.7
18	1.5	3.0	3.0	75.1	1.5	64.4	0.9	73.6	63.5
24	2.5	8.0	8.0	76.3	2.4	74.6	0.8	73.9	73.8
26	2.0	6.0	2.0	63.4	ND	56.3	8.5	63.4	47.8
48	1.3	4.5	2.5	69.0	1.6	63.5	0.9	67.4	62.6
60	50.0	35.0	15.0	73.1	4.8	64.2	0.8	68.3	63.4

^a^ The values are the ng in 50 µL required for 50% inhibition of ^125^I-trastuzumab reactivity with chimeric Erb2/Fc. ^b^ ND, not determined. ^c^ The adjusted % bound values are the % bound minus the % bound when 10 µg of trastuzumab is added to the wells along with the ^212^Pb-labeled trastuzumab.

#### 2.3.3. Immunoreactivity of cGMP ^212^Pb-TCMC-Trastuzumab

The percent of the cGMP ^212^Pb-TCMC-trastuzumab bound to the chimeric Erb2/Fc antigen was as high as 76.3 to a low of 57.8, with no obvious time-dependent pattern being observed (Table 2). When these values were adjusted for non-specific reactivity, demonstrated by adding 10 µg of trastuzumab to one set of wells, the percent bound ranged from 45.2 to 73.9. The adjusted % bound of the Reference ^212^Pb-TCMC-trastuzumab was 47.8 to 73.8.

### 2.4. Effects of Storage at Higher Temperatures on cGMP TCMC-Trastuzumab

During the review and approval process for the IND and clinical trial, members of the FDA panel expressed interest in assays which would identify either degradation products or aggregates in the cGMP TCMC-trastuzumab. To address this query, a study was designed to stress the cGMP TCMC-trastuzumab under conditions that might result in either of the two scenarios. At −3 months, −1 month and −2 weeks, vials (*n* = 2) of the cGMP TCMC-trastuzumab were removed from storage at 4 °C and left at either room temperature or incubated at 37 °C. At T0, the vials were then evaluated using the same assays and techniques described above for the stability studies. The assays also included cGMP TCMC-trastuzumab that was stored at 4 °C, Reference TCMC-trastuzumab, and native trastuzumab. For this particular set of studies the radiolabelings were performed with ^203^Pb (t_1/2_ = 51.9 h), a surrogate for ^212^Pb. ^203^Pb is efficiently produced in cyclotron facilities simplifying the logistics of executing complex experiments. The longer half-life and simpler decay path also simplifies data acquisition and analysis.

Visual inspection of the vials revealed no particulates or turbidity (Table S1, Supplementary materials). Protein concentration of the cGMP TCMC-trastuzumab maintained at 21 °C was 6.18, 5.82 and 5.99 mg/mL at 2 weeks, 1 month and 30 months, respectively. In the same order, the protein concentration for the vials left at 37 °C was 5.76, 5.84 and 5.91 while the concentration for the vial stored at 4 °C was 5.67 mg/mL (Table S1). In addition, no significant change was found in the chelate-to-protein ratio (Table S1) with the exception of the sample that was left at 37 °C for 1 month. This did not seem to be a trend since the sample stored at 37 °C for 3 months was not significantly different than the 4 °C sample.

**Figure 4:**
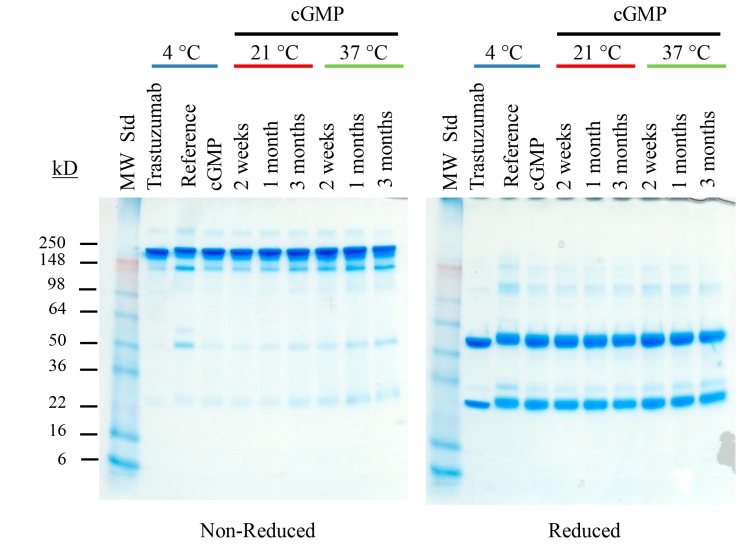
Effect of prolonged storage at elevated temperature on cGMP TCMC-trastuzumab. Vials of cGMP TCMC-trastuzumab were subjected to storage at 21 and 37 °C for 2 weeks, 1 month and 3 months, and then assayed. The SDS-PAGE represents one of the methods assessing integrity of the cGMP TCMC-trastuzumab under non-reduced and reduced conditions.

Integrity of the cGMP TCMC-trastuzumab remained unchanged whether stored at 21 or 37 °C, as determined by SDS-PAGE (Figure 4), SE-HPLC (Tables S2 and S3) or IEX-HPLC (Table S4). When the cGMP TCMC-trastuzumab was labeled with ^203^Pb, a surrogate for the ^212^Pb, the conditions selected for the study did not have an effect on the labeling efficiency or the amount of free chelate in the final product (Table S5). There were also no indications that these stress conditions affected the immunoreactivity of the cGMP TCMC-trastuzumab either before or after labeling with ^203^Pb (Table 3).

A change in the cGMP TCMC-trastuzumab that had been stored at 37 °C for 3 months may be evident in the SE-HPLC analysis of the radiolabeled product. At this condition, only 82.1% and 83% of the radioactivity showed a retention time consistent with the molecular weight of TCMC-trastuzumab (Table 3). Under all of the other conditions tested, at least 98% of the radioactivity was eluted at the appropriate retention time.

**Table 3:** Effect of prolonged storage and elevated temperature on the radiolabeling of cGMP TCMC-trastuzumab with ^203^Pb.

Sample	Temp. (°C)	Time Point	Species	Analysis 1		Analysis 2	
				Retention (min)	%	Retention (min)	%
Reference	4	0	HMW	13.1	1.3	12.8	1.4
			IgG	15.4	91.2	15.4	88.8
			LMW	19.5	6.7	19.4	9.1
cGMP	4	0	HMW				
			IgG	15.7	100	15.8	100
			LMW				
cGMP	21	2 weeks	HMW				
			IgG	15.8	100	15.8	100
			LMW				
cGMP	21	1 month	HMW				
			IgG	15.9	100	15.8	100
			LMW				
cGMP	21	3 months	HMW				
			IgG	16.0	100	15.9	100
			LMW				
cGMP	37	2 weeks	HMW				
			IgG	15.8	98.0	15.7	98.5
			LMW	22.1	2.0	22.1	1.5
cGMP	37	1 month	HMW				
			IgG	15.8	97.7	15.7	98.5
			LMW	22.1	2.3	22.1	1.5
cGMP	37	3 months	HMW				
			IgG	15.8	82.1	15.8	83.0
			LMW	19.6	17.0	19.8	14.0

### 2.5. Effects of Storage at −80 °C as Well as Cycles of Freezing and Thawing on cGMP TCMC-Trastuzumab

**Figure 5:**
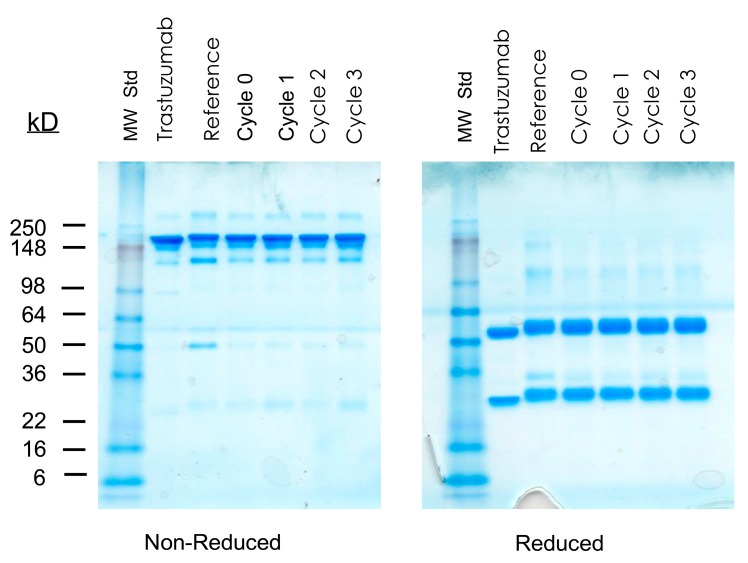
Effect of multiple freeze-thaw cycles on cGMP TCMC-trastuzumab. Vials of cGMP TCMC-trastuzumab were subjected to one, two or three cycles of freezing at −80 °C and thawing. The samples were then assayed. The SDS-PAGE represents one of the methods assessing integrity of the cGMP TCMC-trastuzumab under non-reduced and reduced conditions.

As with the previous temperature stress study, the integrity, immunoreactivity, and thus the stability of the cGMP TCMC-trastuzumab were assessed. Radiolabeling was performed with ^203^Pb and checked for purity and ability to bind with the Erb2/Fc antigen. In brief, storage at −80 °C with up to three freeze/thaw cycles had no measureable effect on the cGMP TCMC-trastuzumab (Tables S6–S13), as compared to the Reference TCMC-trastuzumab or native trastuzumab itself. The SDS-PAGE is presented (Figure 5) to highlight the absence of any affect following the multiple freeze/thaw cycles.

## 3. Discussion

### 3.1. Requirements of Bringing a Radiolabeled Monoclonal Antibody to a Clinical Trial

Providing support for a clinical study requires a substantial commitment of time, dedication of personnel and resources. The financial commitment by Areva Med was considerable to bring ^212^Pb-TCMC-trastuzumab into a clinical trial. Numerous decisions and preparations had to occur in parallel requiring a high level of coordination between the RICS laboratory, Areva Med and the contract GMP facility.

Conjugation of a mAb with a chelate required the development of a family of standard operating protocols (SOPs) using methods that would be used by the manufacturer for the larger scale of production required for clinical trials. The small-scale methods used in the laboratory do not necessarily translate readily to those used in the manufacturing sector [13]. The manufacturer then conducted trial runs conjugating trastuzumab with TCMC to validate the SOP at their facility and to make adjustments. Following each practice, materials were sent back to RICS for validation testing. A singular batch of TCMC was prepared (in the RICS laboratory) for the clinical trial that was also then used for the development of the SOPs and for the trial runs at the GMP manufacturer. A sufficient quantity had to be synthesized, characterized and documented for these purposes. Parallel, a Reference TCMC-trastuzumab conjugate was generated, characterized and documented within the RICS laboratories to be used in the various assays of the cGMP TCMC-trastuzumab.

Prior to the manufacture of the cGMP TCMC-trastuzumab, appropriate tests had to be selected, specifications and acceptance criteria defined, as well as what sampling would be performed. The timeframe for conducting the testing also had to be defined along with adjustments to the list of tests as per the FDA during the IND approval process.

Once the SOPs were validated, production of the cGMP TCMC-trastuzumab moved forward. The bulk material was characterized at both the GMP facility and in the RICS laboratory, and released for vialing. Thereafter, stability testing began with the vialing of the cGMP TCMC-trastuzumab. In all, eleven tests were employed to evaluate the cGMP TCMC-trastuzumab. The tests and data analysis were performed in the RICS laboratory and a Certificate of Analysis provided back to Areva Med.

### 3.2. Evaluation of A GMP mAb, TCMC-Trastuzumab, in Support of a Clinical Trial

The cGMP TCMC-trastuzumab has proven rather stable over the 5 years of evaluation. There were fluctuations in the protein concentration (excepting one time point) and in the molar ratio of chelate to protein, but these were expected experiment/assay variations that were within specifications. No trend was evident between the initial study and the 60 month study. There were no apparent changes in the cGMP TCMC-trastuzumab according to analysis by SDS-PAGE or SE-HPLC. In contrast, the Reference TCMC-trastuzumab has not fared as well. The appearance of a band at ~40 kD, as visualized by SDS-PAGE, that increased with intensity over time suggests that this reference material may be undergoing some form of degradation. The SE-HPLC also supports this suggestion. The percentage of a LMW species increased over time while at the same time there was a decrease in the percentage of the IgG peak. Degradation of the Reference TCMC-trastuzumab may be due to unexplained subtle differences in preparation.

Radiolabeling of the cGMP TCMC-trastuzumab with ^212^Pb also proved consistent throughout the 5 year period. According to any of the criteria by which the ^212^Pb-TCMC-trastuzumab was measured, the labeling efficiency, percent of free chelate, specific activity, immunoreactivity, and purity by SE-HPLC, the integrity of the cGMP TCMC-trastuzumab was not compromised.

Two of the tests indicated that there were changes in the cGMP TCMC-trastuzumab. In one, the IEX-HPLC analysis demonstrated changes that appear to be minimal at this juncture while according to the competition RIA, there may be a decrease in immunoreactivity of the cGMP TCMC-trastuzumab. The charge-based assay, IEX-HPLC, was added to the stability testing per the request of the FDA and was first performed at the 12 month time point. In the first assay, an anomalous peak (Peak 2) was detected at ~2.5 min that represented 1.6% of the product and is material not being retained on the column. At the 60 month time point, this value associated with this peak increased to 2.4%. Peak 2 was also seen in the IEX-HPLC of the Reference TCMC-trastuzumab, although with greater variability in the levels with no discernible trend and only observed twice (0.1%) in IEX-HPLC profiles of the native trastuzumab standard. Ion-exchange chromatography, primarily cation-exchange (CEX), has routinely been used by pharmaceutical companies to monitor biological products. In a study reported by Gandhi *et al*., CEX HPLC was employed to monitor an mAb [14]. The mAb remained unchanged in a liquid formulation during storage at 2–8 °C for a year. However, when maintained at 25 °C, a pre-peak appeared in the CEX-HPLC profile (material not retained by the CEX column), that increased at a rate of 2.5% per month. A rather thorough investigation, which included assessing biological activity of the mAb was then pursued to identify the degradant and the mechanism of the degradation. Using CEX-HPLC, tryptic digestion, and mass spectrometry, the conclusion was that deamidation and clipping of the FcR were the principal sources of the degradants. Whether that is the case for the cGMP TCMC-trastuzumab remains to be answered in future studies. It will be interesting to see if the percentage of Peak 2 increases at the 72 month time point.

The results of the competition RIA are of greater concern as this material moves to any further clinical application. As mentioned, the assay was repeated with a new vial of the cGMP TCMC-trastuzumab and with new preparations of the reagents with a two week interval between the assays. If similar results, or worse yet, the differences between the standards widen, at the next testing, then the cGMP TCMC-trastuzumab will be out of specification.

To date, the data reported herein may represent the most extensive stability testing of a trastuzumab conjugate, albeit modified with the TCMC chelate. Previously, a stability study conducted with trastuzumab diluted at 0.8 and 2.4 mg/mL that indicated the mAb was stable for up to 6 months at either 4 °C or room temperature [15]. In this particular study, CEX-HPLC and SE-HPLC methodologies were employed to detect alterations in trastuzumab. The objective of this study was to actually determine whether Herceptin diluted for a patient dose was stable and could be used for another patient or dose administration in an effort to reduce healthcare costs. Unfortunately and somewhat surprisingly, the immunoreactivity of trastuzumab was not evaluated in that study.

The question of not wasting unused portions of a valuable mAb is a recurring theme in the literature. Papers attesting to the stability of mAbs that have been diluted as they would for a patient dosage and stored for 14 days in a PVC bag are easily found. [15,16,17,18] Invariably, many of these reports and notes only measure one variable to report on the stability of the specific mAb and that measurement tends to be a physical property, *i.e*., protein concentration, which is limited to the detection of the presence of the mAb and not its biological functionality. The possibility of loss of bioactivity, formation of aggregates or degradants apparently seems to not be an important consideration for many investigators and has therefore not been evaluated. Concerns about this deficiency have been expressed and that findings using only one method should be viewed with caution. The use of physical chemical methods certainly does not prove or disprove the stability of a biological product [19]. In fact, to reiterate a point, eleven tests in all were performed on the cGMP TCMC-trastuzumab to demonstrate stability. Six methods were employed to assess integrity while two assays conducted to ascertain retention of the bioactivity of the cGMP TCMC-trastuzumab. It was not until the 5 year testing, that two tests were indicating potential changes in the cGMP TCMC-trastuzumab, one physical (IEX-HPLC) and one for immunoreactivity. Incorporation of multiple methods, not necessarily as many as performed in the studies described herein, to assess a biological product brings a level of confidence to the data that is acquired and subsequent decisions and planning.

### 3.3. Effects of Storage at Higher Temperatures on cGMP TCMC-Trastuzumab

In addition to performing the routine stability studies of the cGMP TCMC-trastuzumab, an experiment was conducted to stress the mAb and then determine which assay best identified the presence of degradants or aggregates. The cGMP TCMC-trastuzumab was stored for 2 weeks, 1 month or 3 months at 21 °C or 37 °C and then subjected to the same tests performed as described above. Interestingly, of all the assays executed, only the SE-HPLC analysis of the radiolabeled cGMP TCMC-trastuzumab indicated that there may have been an effect on the mAb that had been kept at 37 °C for 3 months. Performed in duplicate, only 82% and 83% of the radioactivity was associated with the IgG peak for this sample versus 100% for all of the other samples tested. Further testing will need to be performed to confirm the validity of this result. With the purpose of combining Herceptin therapy with mild hyperthermia, Herceptin was incubated for 1 h at 42 °C and evaluated for the presence of aggregates and retention of immunoreactivity using flow cytometric analysis. Herceptin (trastuzumab) was found to be stable following brief exposures to elevated temperatures [20]. Although only a single method was utilized to assess these two parameters, the investigators reported no change in the stability of the Herceptin.

### 3.4. Effects of Storage at −80 °C as Well as Cycles of Freezing and Thawing on cGMP TCMC-Trastuzumab

The cGMP TCMC-Trastuzumab is currently stored at 2–8 °C. A second study was performed to determine the tolerance of the cGMP TCMC-trastuzumab to storage at −80 °C and subsequent thawing. Vials of cGMP TCMC-trastuzumab were stored at −80 °C, subjected to one, two or three cycles of freezing and thawing. Even after three cycles, no effect on immunoreactivity or integrity was noted. This result is consistent with another study in which the trastuzumab at a lower protein concentration (0.4 mg/mL), was subjected to six freeze-thaw cycles and then evaluated by protein determination, SDS-PAGE and SE-HPLC [21]. Again, the immunoreactivity of the trastuzumab was not evaluated. The advantages of frozen storage are to increase stability and to reduce microbial growth [22]. Unfortunately, protein destabilization and/or aggregation can occur either during storage or during the process of freezing or thawing. Using chemical denaturation and low pH destabilization, a set of persuasive studies were conducted to determine the temperatures at which maximal thermodynamic stability of an mAb and at which cold denaturation occurs [23]. Using two mAbs that only differed in their complementarity determining regions and some flanking amino acids, the authors concluded that these questions must be answered empirically for each mAb. However, they were able to provide some general guidance. First, the maximal thermodynamic stability of a mAb occurs at ambient temperature. Second, storage at −70 °C to −80 °C is more appropriate than −20 °C. At the lower temperatures, the risk of cold denaturation and formation of aggregates is greatly decreased due to the slower kinetics.

## 4. Experimental Section

### 4.1. Chelate Synthesis and mAb Conjugation

The synthesis, characterization, and purification of the bifunctional ligand 1,4,7,10-tetraaza-1,4,7,10-tetra-(2-carbamoyl methyl)-cyclododecane (TCMC) has been previously described [24]. Trastuzumab (Herceptin^®^, Genentech, Inc., South San Francisco, CA, USA), was purchased through the NIH, Division of Veterinary Resources Pharmacy. Conjugation of trastuzumab (1.2 g) with TCMC was performed at Goodwin Biotechnology, Inc. (Plantation, FL, USA) by established methods using a 20-fold molar excess of ligand to mAb as previously reported [7]. The concentration of trastuzumab prior to and after conjugation was quantified by the method of Lowry using a BSA standard [11]. The Reference TCMC-trastuzumab was prepared in the RICS laboratory following the same SOP developed for the GMP manufacturer albeit at a smaller scale. The RICS preparation was performed with 200 mg of trastuzumab with a 20-fold excess of the TCMC chelate. The final formulation for the cGMP TCMC-trastuzumab was 5.25 mg/mL in 0.15 M ammonium acetate (pH 7.0) while the RICS Reference TCMC-trastuzumab was 3.45 mg/mL in 0.15 M ammonium acetate (pH 7.0).

### 4.2. Arsenazo Assay

The average number of TCMC molecules linked to trastuzumab was determined using a spectrophotometric-based assay [25]. Briefly, a stock solution of Pb(II)-AA(III) in 0.15 M NH_4_OAc buffer, pH 7.0 containing 10 mM AAIII and 4.83 mM Pb(II) was prepared. A titration of cGMP TCMC-trastuzumab was performed by adding 2.5 µL to 900 µL of Pb(II)-AA(III) complex at room temperature. The absorbance at 656 nm was recorded after 15 min. The addition of 2.5 µL cGMP TCMC-trastuzumab was repeated three more times. The mole amount of TCMC was then calculated by dividing the change in absorbance by the extinction coefficient; determined by plotting the change in absorbance vs. assay volume. The chelate:protein (C/P) ratio was then calculated by dividing the molarity of chelate by the molarity of trastuzumab. Each C/P value reported is an average of two assays.

### 4.3. SDS-Polyacrylamide Gel Electrophoresis

The integrity and purity of cGMP TCMC-trastuzumab was evaluated by SDS-PAGE. Five µg of each protein sample was loaded onto a 5%–20% Tris-glycine gel after heating at 100 °C for 3 min in the absence of or presence of β-mercaptoethanol (non-reduced and reduced, respectively). The sample was compared to a reference TCMC-trastuzumab prepared at the RICS, unmodified trastuzumab and a molecular weight standard.

### 4.4. Radiolabeling

#### 4.4.1. Radio-Iodination with ^125^I

Radio-iodination of trastuzumab with ^125^I, required for the competition RIA, was performed using Iodo-Gen (Pierce Chemical, Rockford, IL, USA) [26]. Briefly, a solution of Na^125^I (0.5–1 mCi) was added to a solution of trastuzumab (50 μg) in phosphate buffer (100 μL, 0.1 M, pH 7) contained in a test tube pre-coated with Iodo-Gen and incubated at ambient temperature for 3 min. The radio-iodinated product was purified using a PD-10 (GE Healthcare, Piscataway, NJ, USA) desalting column.

#### 4.4.2. Radiolabeling with ^212^Pb

The initial validation of the vialed cGMP TCMC-trastuzumab and subsequent stability studies were performed with ^212^Pb. The ^212^Pb, eluted from ^224^Ra generators supplied by Areva Med LLC (Bethesda, MD, USA) has been previously described [12]. Briefly, the ^224^Ra generator (2–5 mCi) was washed (2 M HCl) to remove impurities, unbound ^224^Ra, daughter isotopes, organic residue or any damaged resin. On the following day, ^212^Pb was eluted from the generator with 2 M HCl. The eluate was heated to dryness, digested three times with nitric acid (8 M) and the dry residue dissolved in 0.1 M HNO_3_. TCMC-trastuzumab was then labeled with ^212^Pb as previously described [7,12]. Radioactivity measurements were performed using a calibrated Ge(Li) detector (model GEM10185-P; EG&G Ortec, Oak Ridge, TN, USA) coupled to a multichannel analyzer and a computer running Gamma Vision version 5.2 software (EG&G Ortec). The ^212^Pb activity was determined by measurement of the 238.6 keV γ-ray.

#### 4.4.3. Radiolabeling with ^203^Pb

The additional testing of the cGMP TCMC-trastuzumab was performed using ^203^Pb, produced from a cyclotron by a ^203^Tl(d,n)^203^Pb reaction as previously described [27]. After irradiation, the target was dissolved by boiling in HNO_3_, the solution evaporated to dryness and the process repeated to ensure that the target is completely dissolved. The final residue was re-dissolved in HNO_3_ and applied to a pre-conditioned Pb resin (Eichrom Technologies Inc., Lisle, IL, USA). Resin preconditioning was performed by successive washing with HNO_3_ at 6 M, 3 M, 1 M, 0.1 M, 0.01 M and 1 M. Tl was washed off the column with HNO_3_ (1 M), monitored by following the initial radioactivity (^202^Tl) eluted from the column using a Capintec Radioisotope Calibrator (Model CRC-127R) at the 344 calibration setting. After the radioactivity levels returned to background, ^203^Pb was eluted with 0.01 M HNO_3_. The combined ^203^Pb eluate was evaporated to dryness and the residue re-dissolved in 0.1 M HNO_3_ for subsequent labeling. The ^203^Pb solution was neutralized and the cGMP TCMC-trastuzumab (100 µg) added, vortexed and incubated at 37 °C for 1 h. The reaction was quenched with EDTA solution (0.1 M, 4 µL) and the radiolabeled cGMP TCMC-trastuzumab purified using a PD-10 desalting column (GE Healthcare).

### 4.5. Instant Thin Layer Chromatography (ITLC)

ITLC was performed on the labeling reaction mixture and the purified cGMP TCMC-trastuzumab. The stationary phase for ITLC was ITLC^TM^ SG (Varian Inc., Lake Forest, CA, USA). Two mobile phases were employed: (A) 10 mM EDTA in 0.15 M NH_4_OAc and (B) 10 mM NaOH in normal saline. Two 15 mL polypropylene (PP) tubes per sample were labeled Solvent A and Solvent B, containing1 mL of the respective mobile phase solution. The ITLC sheets were cut into 1 × 10 cm strips, marked 1 cm from one end to indicate the solvent front, and 1.5 cm from the other end to indicate the origin. Two ITLC strips each were spotted at the origin with 1–2 μL of test ^203^Pb^2+^ or ^212^Pb^2+^ solution, quenched reaction mixture, or PD-10 purified radioimmunoconjugate. One strip from each pair was inserted into a PP tube labeled Solvent A and the other strip was inserted into a PP tube labeled Solvent B making sure that the end marked for the origin touches the solvent, but the spot was above the solvent. After the solvent reached the solvent front, the strips were transferred to an appropriately labeled empty 15 mL PP tube. After 5 min, the ITLC strips were cut in half and each half was placed in a 20 mL scintillation vial and counted in a γ-scintillation counter (Wizard One, PerkinElmer, Shelton, CT, USA). The percent (%) count at the origin half and solvent front half was calculated. The % free ^203^Pb or ^212^Pb was determined from the radioactivity at the solvent front of the ITLCs in solvent A. The % radiochemical yield was determined from the radioactivity at the origin for the quenched reaction mixture. The purity of the product was determined from the radioactivity at the origin for the PD-10 purified product sample.

### 4.6. Radioimmunoassays

#### 4.6.1. Immunoreactivity of cGMP TCMC-Trastuzumab Assessed by Competition Radioimmunoassay

The immunoreactivity of cGMP TCMC-trastuzumab was evaluated in a competition RIA. Briefly, 10 ng of chimeric Erb2/Fc (R&D Systems, Minneapolis, MN, USA) were added to the wells of a 96-well plate. Following an overnight incubation at 4 °C, wells were aspirated and 150 µL of PBS/BSA added to each well. After l h at ambient temperature, the wells were aspirated and serial dilutions (3 ng to 3 µg in 50 µL BSA/PBS) of native (unmodified) trastuzumab, cGMP TCMC-trastuzumab and the Reference TCMC-trastuzumab were added to the wells in triplicate, one set of wells received BSA/PBS without any competitor, along with ^125^I-trastuzumab (~25,000 cpm in 50 µL BSA/PBS). The wells were aspirated after 2 h at 37 °C and washed with BSA/PBS. The bound radioactivity was removed with 100 µL 0.2 M NaOH, adsorbed to cotton filters, placed in 12 × 75 mm tubes and counted in a γ-scintillation counter. The percent inhibition was calculated using the buffer control and plotted. HuM195, an anti-CD33 mAb provided by Dr. M. McDevitt, Memorial Sloan-Kettering Cancer Center, served as a negative control.

#### 4.6.2. Immunoreactivity of Radiolabeled TCMC-Trastuzumab

The immunoreactivity of the ^212^Pb- or ^203^Pb-trastuzmab was assessed in a RIA with 50 ng of chimeric Erb2/Fc adsorbed to the wells of a 96-well plate. After blocking of the wells with BSA/PBS, serial dilutions of the radiolabeled trastuzumab (~300,000 to ~12,500 cpm in 50 µL of BSA/PBS) were added to the wells and incubated for 4 h at 37 °C. The wells were then washed, the radioactivity desorbed as described above and counted in a γ-scintillation counter. The percentage binding was calculated for each dilution and averaged. The specificity of the radiolabeled trastuzumab was confirmed by incubating one set of cells with radiolabeled trastuzumab and 10 μg of unlabeled trastuzumab.

### 4.7. High Performance Liquid Chromatography (HPLC)

#### 4.7.1. Size-Exclusion (SE) HPLC

The integrity of the cGMP TCMC-trastuzumab as well as the radiolabeled products were evaluated by size-exclusion (SE) high performance liquid chromatography (HPLC) using an analytical TSK-3000SW column (Tosoh Bioscience, Montgomeryville, PA, USA). The test samples (50 µg) were eluted at a flow rate of 0.5 mL/min with 100 mM potassium chloride in 67 mM sodium phosphate (pH 6.8). The TCMC-trastuzumab conjugate was detected at 280 nM (~15 min). ^212^Pb-TCMC-trastuzumab was detected at ~15.8 min by collecting fractions and measuring the radioactivity in a γ-scintillation counter.

#### 4.7.2. Ion-Exchange (IEX) HPLC

The cGMP TCMC-trastuzumab was also analyzed in a charge-based assay using ion-exchange (IEX) HPLC. The cGMP TCMC-trastuzumab (150 µg) was applied to a strong anion exchange column for 5 min (Zorbax SAX, Agilent, Santa Clara, CA, USA) equilibrated in 10 mM sodium phosphate (pH 7.2) and eluted at a flow rate of 1 mL/min using a gradient of 0%–100% 1 M NaCl over 15 min. The protein was detected at 280 nm with a retention time of 9.8 min.

## 5. Conclusions

In summary, the immunoconjugate, the TCMC-trastuzumab, has proven to be a robust construct. Eleven tests were selected and performed on the cGMP TCMC-trastuzumab at pre-vialing, vialing, 3, 6, 12, 18, 24, 36, 48 and 60 months; the stability testing was performed 10 times over a 5 year period. To date, only two tests have indicated the possibility of product failure. The charge-based assay (IEX-HPLC) and an immunoreactivity assay (competition RIA) have shown changes in the cGMP TCMC-trastuzumab. Subsequent testing will determine if there is a trend to these results and the product has become compromised, or if this is simply experimental variance. The additional testing, exploring the tolerance of cGMP TCMC-trastuzumab at higher temperatures and multiple freeze-thaw cycles attests to the stability of the immunoconjugate. Considering the resources (financial, time and personnel) required to translate a radioimmunoconjugate from the bench to bedside through to treating patients, these results are certainly encouraging.

## Supplementary Files

Supplementary File 1

## Abbreviations

RIT: radioimmunotherapy
UAB: University of Alabama at Birmingham
TCMC: 1,4,7,10-tetraaza-1,4,7,10-tetra-(2-carbamoylmethyl)-cyclododecane
cGMP: current good manufacturing practices
IEX-HPLC: ion-exchange high performance liquid chromatography
RIA: radioimmunoassay
SE-HPLC: Size-exclusion high performance liquid chromatography
mAb: monoclonal antibody
FDA: Food and Drug Administration
CRADA: Collaborative reasearch and development agreement
RICS: Radioimmune & Inorganic Chemistry Section
IND: Investigational new drug
IHC: immunohistochemistry
IC: immunoconjugate
BSA: bovine serum albumin
C/P: chelate-to-protein ratio
SDS-PAGE: sodium dodecyl sulfate-polyacrylamide gel electrophoresis
IgG: immunoglobulin
SOPs: standard operating protocols
