# Supplementary material for: Bench to Bedside: Stability Studies of GMP Produced Trastuzumab-TCMC in Support of a Clinical Trial
Source: Pharmaceuticals (Basel). 2015 Jul 29;8(3):435–54. doi: 10.3390/ph8030435 (PMC4588176; doi:10.3390/ph8030435)
Supplement: Supplementary File 1 [file pharmaceuticals-08-00435-s001.pdf]

Supplementary Materials

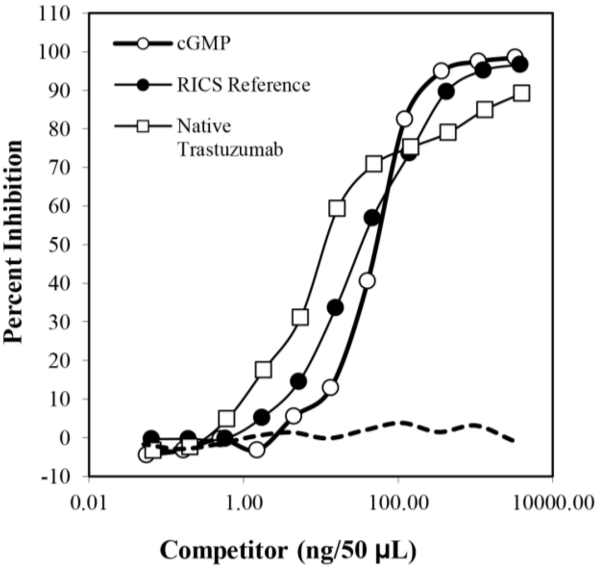

50% Inhibition	ng/50 μL
cGMP	50
RICS Reference	35
Native Trastuzumab	10

Figure S1. 60 month evaluation of trastuzumab-TCMC (339093-FVP) immunoreactivity by competition radioimmunoassay.

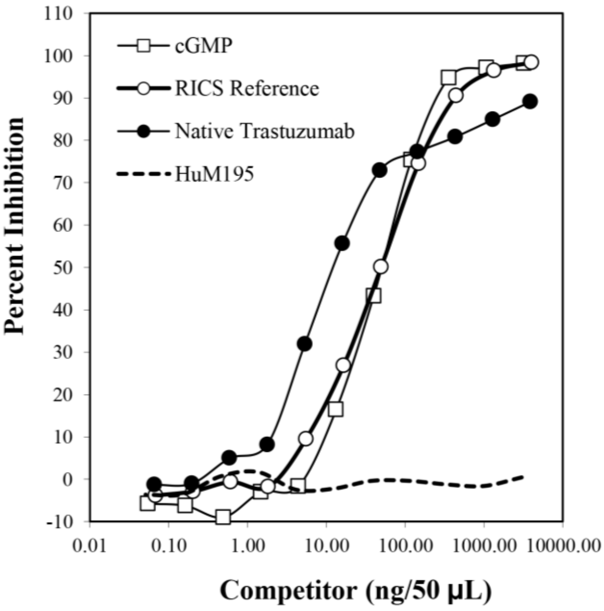

50% Inhibition	ng/50 μL
cGMP	50
RICS Reference	50
Native Trastuzumab	15

Figure S2. Repeat 60 month evaluation of cGMP trastuzumab-TCMC immunoreactivity by competition radioimmunoassay.

Table S1. Effect of prolonged storage and elevated temperature on cGMP TCMC-trastuzumab.

Sample	Condition (°C)	Time	Assay				
			Visual Inspection		Lowry	Arsenazo	
			Clear	Particulates	mg/mL	C/P Ratio	*P* Value
cGMP	4	0	Yes	No	5.67	12.2 ± 2.0	
cGMP	21	2 weeks	Yes	No	6.18	10.6 ± 2.1	0.245 [a]
cGMP	21	1 month	Yes	No	5.82	12.8 ± 1.7	0.513
cGMP	21	3 months	Yes	No	5.99	10.7 ± 1.1	0.123
cGMP	37	2 weeks	Yes	No	5.76	12.7 ± 3.2	0.141
cGMP	37	1 month	Yes	No	5.84	14.1 ± 2.2	0.017
cGMP	37	3 months	Yes	No	5.91	13.8 ± 4.2	0.136

[a] The Student T test was performed, each condition is compared to the cGMP TCMC-trastuzumab maintained at 4 °C.

Table S2. Effect of prolonged storage and elevated temperature on the integrity of cGMP TCMC-trastuzumab using SE-HPLC.

Sample	Temp. (°C)	Time Point	Species	Analysis 1		Analysis 2	
				Retention (min)	% Area	Retention (min)	% Area
			HMW	12.91	0.23	12.82	0.20
Trastuzumab	4	---	IgG	15.65	99.77	15.56	99.80
			LMW	0	0	0	0
			HMW	13.61	2.77	13.35	4.62
Reference	4	---	IgG	16.30	97.10	16.49	90.18
			LMW	0	0	0	5.21
			HMW	13.82	2.00	13.88	1.19
cGMP	4	---	IgG	17.11	89.44	17.35	88.22
			LMW	21.40	8.39	21.48	10.59
			HMW	13.88	1.20	14.12	0.69
cGMP	21	2 weeks	IgG	17.10	90.59	17.42	90.90
			LMW	21.54	8.25	21.52	8.41
			HMW	13.88	0.98	14.13	0.69
cGMP	21	1 month	IgG	17.07	91.37	17.43	92.18
			LMW	21.55	7.66	21.42	7.14
			HMW	13.68	0.81	14.00	0.69
cGMP	21	3 months	IgG	17.10	91.77	17.41	91.37
			LMW	21.58	7.43	22.25	7.94
			HMW	13.71	1.20	13.94	0.98
cGMP	37	2 weeks	IgG	17.08	92.29	17.44	92.74
			LMW	21.52	6.51	22.07	6.27
			HMW	13.83	0.86	14.14	0.51
cGMP	37	1 month	IgG	17.13	91.78	17.45	91.46
			LMW	21.55	7.35	21.40	8.04
			HMW	13.71	1.16	14.02	0.70
cGMP	37	3 months	IgG	17.12	92.20	17.45	90.19
			LMW	21.46	6.64	22.10	9.12

Table S3. Gel filtration Standard (Biorad 151-1901).

Component	MW (kD)	Retention Time (min)	
		Analysis 1	Analysis 2
Thyroglobulin	670	11.31	11.41
γ-globulin (bovine)	158	15.55	15.82
Ovalbumin (chicken)	44	17.40	17.53
Myoglobin (horse)	17	20.72	21.01
Vitamin B12	1.35	25.11	25.45

Table S4. Effect of prolonged storage and elevated temperature on the integrity of cGMP TCMC-trastuzumab using IEX-HPLC.

Sample	Temp. (℃)	Time point	Species (min)	Analysis 1		Analysis 2	
				Retention (min)	% Area	Retention (min)	% Area
			2	----	----	----	----
Trastuzumab	4	0	IgG	10.09	99.47	10.12	98.50
			3	----	----	----	----
			2	2.37	0.15	2.25	0.22
Reference	4	0	IgG	10.00	89.72	10.05	90.89
			3	11.73	8.98	11.633	6.96
			2	2.17	2.10	2.21	1.94
cGMP	4	0	IgG	9.67	97.27	9.83	96.95
			3	----	----	14.55	0.16
			2	2.18	2.04	2.26	2.12
cGMP	21	2 weeks	IgG	9.77	97.07	9.80	96.18
			3	14.14	0.22	14.49	0.29
			2	2.18	1.95	2.26	2.10
cGMP	21	1 month	IgG	9.75	97.22	9.78	96.50
			3	14.11	0.25	14.54	0.25
			2	2.17	2.46	2.26	2.43
cGMP	21	3 months	IgG	9.78	96.28	9.83	95.90
			3	14.30	0.44	14.52	0.33
			2	2.19	2.34	2.27	2.39
cGMP	37	2 weeks	IgG	9.80	96.81	9.85	95.83
			3	14.22	0.45	14.60	0.44
			2	2.19	2.68	2.25	2.64
cGMP	37	1 month	IgG	9.85	95.83	9.83	95.14
			3	14.35	0.65	14.47	0.83
			2	2.19	2.99	2.26	2.99
cGMP	37	3 months	IgG	9.90	94.10	9.91	93.79
			3	14.37	1.93	14.59	1.79

Peak 2 ~2.2 min; Peak 1 (IgG) ~9.8 min; Peak 3 ~14 min.

Table S5. Effect of prolonged storage and elevated temperature on the radiolabeling of cGMP TCMC-trastuzumab.

Sample	Condition ((°C)	Timepoint	% Free Chelate	% Efficiency	Specific Activity (mCi/mg)
Reference	4	0	0.2	97.6	10.6
cGMP	4	0	1.7	96.7	9.3
cGMP	21	2 weeks	0.2	97.4	7.4 [a]
cGMP	21	1 month	0.1	98.0	7.5 [a]
cGMP	21	3 months	0.2	97.7	7.2 [a]
cGMP	37	2 weeks	1.1	96.5	8.4
cGMP	37	1 month	1.4	96.2	9.7
cGMP	37	3 months	1.6	96.2	8.9

[a] The differences in the specific activity for the samples maintained at 21 °C is due to a lower yield of ^{203}Pb recovered from the cyclotron target. Due to the number of samples, two targets were processed for radiolabeling of the samples, one week apart.

Table S6. Effect of prolonged storage and elevated temperature on the immunoreactivity of cGMP TCMC-trastuzumab.

Sample	Condition (°C)	Time	Assay			
			Competition	Direct Binding		
			50% Inhibition (ng/50 μL)	% Specific	% NonSpecific	Corrected %
Trastuzumab	4		3.0			
Reference	4	0	6.0	63.7	3.0	60.7
cGMP	4	0	2.1	69.1	1.5	67.6
cGMP	21	2 weeks	2.8	65.0	1.1	63.9
cGMP	21	1 month	2.8	68.2	2.3	65.9
cGMP	21	3 months	2.8	63.7	3.6	60.1
cGMP	37	2 weeks	2.8	68.7	1.2	67.5
cGMP	37	1 month	2.0	67.7	1.1	66.6
cGMP	37	3 months	2.8	62.7	1.0	61.7

Table S7. Effect of multiple freeze-thaw cycles on cGMP TCMC-trastuzumab.

Sample	Freeze/Thaw Cycle	Assay				
		Visual Inspection		Lowry	Arsenazo	
		Clarity	Particulates	mg/mL	C/P Ratio	*P* Value
cGMP	0	Clear	No	5.07	12.8 ± 1.5	
cGMP	1	Clear	No	5.13	12.0 ± 0.7	0.303 [a]
cGMP	2	Clear	No	5.15	13.1 ± 1.8	0.690
cGMP	3	Clear	No	5.43	12.9 ± 1.5	0.833

[a] The Student T test was performed, each condition is compared to the cGMP TCMC-trastuzumab maintained at 4 °C.

Table S8. Effect of multiple freeze-thaw cycles on the integrity of cGMP TCMC-trastuzumab analyzed by SE-HPLC.

Sample	Freeze/Thaw Cycle	Species	Analysis 1		Analysis 2	
			Retention (min)	%	Retention (min)	%
Trastuzumab	0	HMW	12.77	0.18	12.73	0.20
		IgG	15.37	99.68	15.34	99.74
		LMW	0	0	0	0
Reference	0	HMW	13.46	2.48	13.46	3.19
		IgG	16.10	91.29	16.11	89.99
		LMW	20.30	6.23	20.30	6.82
cGMP	0	HMW	13.99	2.25	13.89	1.77
		IgG	17.33	91.5	16.94	90.84
		LMW	20.36	4.2	21.63	5.46
cGMP	1	HMW	14.30	1.31	13.63	1.01
		IgG	17.54	92.05	16.92	92.83
		LMW	21.75	5.04	21.68	3.91
cGMP	2	HMW	14.36	1.12	13.82	0.98
		IgG	17.53	92.15	16.92	93.39
		LMW	21.82	4.48	21.75	3.98
cGMP	3	HMW	14.16	0.98	13.82	1.77
		IgG	17.45	92.63	16.91	93.15
		LMW	21.68	4.21	22.17	3.46

Table S9. Gel filtration Standard (Biorad 151-1901).

Component	MW (kD)	Retention Time (min)	
		Analysis 1	Analysis 2
Thyroglobulin	670	11.12	11.19
γ-globulin (bovine)	158	15.36	15.42
Ovalbumin (chicken)	44	17.28	17.34
Myoglobin (horse)	17	20.60	20.64
Vitamin B12	1.35	24.859	24.85

Table S10. Effect of multiple freeze-thaw cycles on the integrity of cGMP TCMC-trastuzumab analyzed by IEX-HPLC.

Sample	Freeze/Thaw Cycle	Species	Analysis 1		Analysis 2	
			Retention (min)	%	Retention (min)	%
Trastuzumab	0	1	0	0	0	0
		IgG	10.04	97.04	10.06	97.62
		3	12.00	1.34	12.00	0.97
Reference	0	1	2.35	0.23	2.37	0.08
		IgG	9.81	94.13	9.81	94.57
		3	12.00	4.54	12.02	4.28
cGMP	0	1	2.29	0.93	2.27	1.02
		IgG	9.54	97.72	9.52	97.37
		3	12.00	0.30	12.00	0.61
cGMP	1	1	2.23	1.04	2.24	1.01
		IgG	9.59	97.46	9.63	97.39
		3	12.0	0.51	12.00	0.62
cGMP	2	1	2.24	0.97	2.24	0.97
		IgG	9.58	97.17	9.59	97.58
		3	12.00	0.21	12.00	0.52
cGMP	3	1	2.21	1.01	2.28	0.98
		IgG	9.62	97.25	9.62	97.32
		3	12.00	0.76	12.00	0.73

Table S11. Effect of multiple freeze-thaw cycles on the radiolabeling of cGMP TCMC-trastuzumab.

Sample	Freeze/Thaw Cycle	% Free Chelate	% Efficiency	Specific Activity (mCi/mg)
Reference	0	0.4	99.4	12.1
cGMP	0	0.4	99.6	10.1
cGMP	1	0.3	93.5	10.3
cGMP	2	0.3	99.5	9.9
cGMP	3	0.2	99.5	9.9

Table S12. Effect of multiple freeze-thaw cycles on the immunoreactivity of cGMP TCMC-trastuzumab.

Sample	Freeze/Thaw Cycle	Assay			
		Competition	Direct Binding		
		50% Inhibition (ng/50 µL)	% Specific	% Non-Specific	Corrected %
Trastuzumab	0	8			
Reference	0	12	74.4	8.1	66.3
cGMP	0	3.5	81.1	12.3	68.8
cGMP	1	4.0	69.8	12.8	57.0
cGMP	2	5.8	72.5	14.4	57.1
cGMP	3	5.8	76.3	10.3	66.0

Table S13. Effect of multiple freeze-thaw cycles on the integrity of radiolabeled of cGMP TCMC-trastuzumab.

Sample	Freeze/Thaw Cycle	Species	Analysis 1		Analysis 2	
			**Retention (min)**	**%**	**Retention (min)**	**%**
Reference	0	HMW	12.7	2.3	12.8	1.9
		IgG	15.1	89.9	15.2	88.2
		LMW	19.5	7.8	19.5	8.1
cGMP	0	HMW<br>IgG<br>LMW	15.6	100	15.6	100
cGMP	1	HMW<br>IgG<br>LMW	15.6	100	15.5	100
cGMP	2	HMW<br>IgG<br>LMW	15.6	100	15.6	100
cGMP	3	HMW<br>IgG<br>LMW	15.5	100	15.6	100

© 2015 by the authors; licensee MDPI, Basel, Switzerland. This article is an open access article distributed under the terms and conditions of the Creative Commons Attribution license (http://creativecommons.org/licenses/by/4.0/).
